# Modulating Cognitive–Motor Multitasking with Commercial-off-the-Shelf Non-Invasive Brain Stimulation

**DOI:** 10.3390/brainsci12020180

**Published:** 2022-01-29

**Authors:** Nathan Ward, Erika Hussey, Thomas Wooten, Elizabeth Marfeo, Tad T. Brunyé

**Affiliations:** 1 Department of Psychology, Tufts University, Medford, MA 02155, USA; tom.wooten16@gmail.com; 2 Defense Innovation Unit, Mountain View, CA 94043, USA; erikahussey@gmail.com; 3 Department of Occupational Therapy, Tufts University, Medford, MA 02155, USA; elizabeth.marfeo@tufts.edu; 4 U.S. Army DEVCOM Soldier Center, Natick, MA 01760, USA; tbruny01@tufts.edu; 5 Center for Applied Brain & Cognitive Sciences, Tufts University, Medford, MA 02155, USA

**Keywords:** cognitive–motor dual-tasking, tDCS, multitasking

## Abstract

One growing area of multitasking research involves a focus on performing cognitive and motor tasks in tandem. In these situations, increasing either cognitive or motor demands has implications for performance in both tasks, an effect which is thought to be due to competing neural resources. Separate research suggests that non-invasive brain stimulation may offer a means to mitigate performance decrements experienced during multitasking. In the present study, we investigated the degree to which a commercially available non-invasive brain stimulation device (Halo Sport) alters balance performance in the presence of different types of cognitive demands. Specifically, we tested if performing a secondary cognitive task impacts postural sway in healthy young adults and if we could mitigate this impact using transcranial direct current stimulation (tDCS) applied over the primary motor cortex. Furthermore, we included conditions of unstable and stable surfaces and found that lower surface stability increased postural sway. In addition, we found that cognitive load impacted postural sway but in the opposite pattern we had anticipated, with higher sway found in the single-task control condition compared to executive function conditions. Finally, we found a small but significant effect of tDCS on balance with decreased sway for active (compared to sham) tDCS.

## 1. Introduction

For many decades, researchers thought that maintaining balance was an entirely automatic task; however, we now have strong evidence that standing upright involves attention [1,2]. Indeed, postural control involves interactions between cortical and cerebellar regions [3], as well as interactions among fronto-striatal regions [4]. Further evidence comes from cognitive–motor multitasking paradigms that focus on costs when combining cognitive and postural tasks [5,6,7]. These costs may stem from competition for attentional resources [8,9] or from bottlenecks in our information-processing architecture [10]. In addition, cognitive–motor multitasking may rely on higher cognitive processes, including emotion regulation [11]; perceptual–cognitive changes during motor learning [12]; and, most importantly, for the current study, executive functions [13,14]. In healthy young adults, the magnitude of cognitive–motor multitasking costs varies according to both motor and cognitive demands [15], and often we see even greater interference in older adults [16] and on unstable surfaces [17,18], especially when texting while standing upright [19].

Given the importance of balance for everyday life, we focus on ways to reduce cognitive–motor multitasking costs. One approach involves transcranial direct current stimulation, or tDCS. tDCS is an affordable and portable form of neuromodulation that is safe [20] and has been used in a number of basic and applied studies targeting cognitive [21,22,23] and motor functioning [24,25,26]. Although the exact mechanisms are still being uncovered, tDCS modulates cortical excitability via weak direct currents applied to the scalp with two or more electrodes [27,28,29]. tDCS has already been used to successfully target cognitive multitasking [30,31]. Most importantly for the current research, tDCS has also been used to target cognitive–motor multitasking performance.

For example, in one study 20 healthy adults completed single- and dual-task gait and postural control scenarios before and after receiving active or sham tDCS targeting the left dorsolateral prefrontal cortex [32]. Active tDCS improved postural control only when a secondary cognitive demand (i.e., serial subtraction) was present. In a different study, 10 healthy adults completed single- and dual-task balance scenarios in different stances before and after receiving active tDCS to the dorsolateral prefrontal cortex or the supplementary motor area [33]. In contrast to the prior study, there were no significant impacts of either tDCS intervention on cognitive–motor multitasking performance. Given these conflicting results, more research is needed to better understand how and when tDCS can be used to impact cognitive–motor multitasking. 

In the current study, we compared the impact of active vs. sham tDCS over motor cortex on postural sway during single- and dual-task scenarios (and on stable vs. unstable surfaces). This differs from the two prior studies on tDCS and cognitive–motor multitasking with healthy adults in several ways. First, participants received stimulation in single- and dual-task balance scenarios (i.e., online) rather than before and after (i.e., offline). We based this decision on prior research that found superior effects for online (vs. offline) tDCS in cognitive and motor learning [34,35]. Second, we explicitly targeted the primary motor cortex rather than the dorsolateral prefrontal cortex to investigate whether it was possible to impact the motor aspect of cognitive–motor multitasking. Indeed, prior research has found improvements in postural control with tDCS over motor cortex in single-task scenarios [36,37], and we sought to extend this to dual-task scenarios that include cognitive demands. Third, we used four different types of secondary cognitive dual-tasks to understand the degree to which cognitive–motor multitasking depends on the nature of the cognitive demand: three executive function (EF) tasks (shifting, updating, and inhibition) and one non-EF task (processing speed). We based this decision on prior research that found that the interaction between cognitive and postural tasks depends on the level of cognitive load [38,39], as operationalized by the kind of executive function engaged. Furthermore, by including both non-EF and EF tasks, we could investigate whether balance was impacted by a secondary cognitive task regardless of any specific EF demands or if EF tasks would be more difficult than the non-EF task and thus lead to greater impairments in postural control [40]. Finally, we utilized a commercial-off-the-shelf (COTS) neurostimulation device, the Halo Sport, to deliver electrical current over primary motor cortex. To our knowledge, this is the first study to use a COTS solution within the context of cognitive–motor multitasking. 

Prior to any data collection, we preregistered our study design and predictions online: https://osf.io/uq5b7. To briefly summarize, we tested the impact of COTS tDCS delivered over the motor cortex on cognitive–motor multitasking by manipulating cognitive (during single-task scenarios and 4 dual-task scenarios) and balance demands (on stable vs. unstable surfaces). Balance was assessed using sway metrics from IMUs and cognitive task performance was assessed on each of the secondary tasks using response time or accuracy.

Among the balance measures, we predicted that postural sway would be greater under situations with higher cognitive and balance demands and that the intersection of these demands would generate the greatest degree of sway variability. Specifically, we expected sway to be more pronounced on unstable surfaces relative to stable surfaces and to be greater under dual-task scenarios relative to a single task [41,42,43]. We expected active tDCS to mitigate these sway effects compared to sham tDCS, such that tDCS would decrease sway under conditions of heightened cognitive and balance demands. Finally, we sought to explore the impacts of different kinds of secondary dual tasks by comparing sway when participants stood on surfaces of varying stability and completed non-EF (processing speed) vs. EF (shifting, updating, and inhibition). We expected sway to be greater when EF was required, in part because a limited pool of neural resources is thought to subserve both the EF tasks and the coordination of cognitive–motor multitasking [44]. 

In terms of cognitive task performance, we anticipated a poorer performance (slower response times and lower accuracy) in the presence of greater balance demands (i.e., completing the tasks while standing on unstable surfaces relative to stable surfaces). We expected these effects to decrease when active tDCS was delivered relative to the sham condition, such that administering tDCS would provide protective benefits against latency slowing or inaccurate responding under balance demands.

## 2. Materials and Methods

### 2.1. Participants

We recruited 80 healthy young adults (M_age_ = 20.8 SD_age_ = 3.4 | 51 females and 29 males) for this study and compensated them $15 for their hour of participation. Two participants withdrew from the study early and we discarded their data. We selected our sample size to be at least double the sample size of two prior studies that used the same COTS device to target physical performance [45,46]. Exclusion criteria included if participants had any metallic implants in the head; implanted internal or external electrical stimulation devices; previous adverse reactions to tDCS or other forms of low-current brain stimulation; previous seizures, head injuries, or other brain-related conditions; previous illnesses that had caused brain injury; any diagnoses of a neurological or psychiatric disorder; or a sensitive scalp. All participants provided informed consent according to the Declaration of Helsinki and ethics approval granted by the Tufts University Institutional Review Board (#1611020 approved 14 December 2016).

### 2.2. Protocol

**Figure 1:**
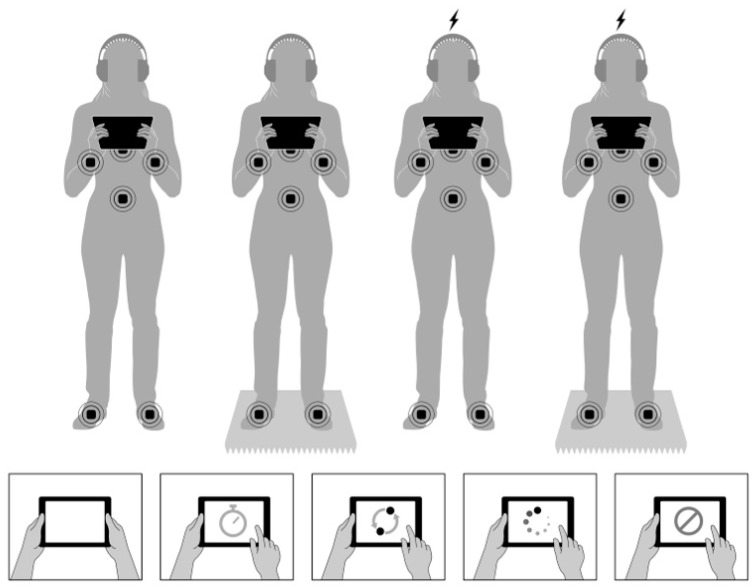
Top figures represent sensor placement in the Active tDCS (**top right**) and Sham tDCS (**top left**) conditions as participants performed the various conditions on stable and unstable surfaces. Bottom images depict the five experimental conditions, which were baseline (no task), non-EF processing speed, EF shifting, EF updating, and EF inhibition.

Participants completed one study visit that lasted approximately one hour. After the participants provided informed consent, we randomized them into one of two stimulation groups: active vs. sham. Both participants and experimenters were blinded to the stimulation assignment. Next, we fitted participants with wearable inertial measurement unit (IMU) sensors that measured postural sway and a neuromodulation device that delivered non-invasive brain stimulation. Participants then completed a standing baseline condition on both firm and foam surfaces while holding a tablet before completing four cognitive tasks twice via the tablet: once on a firm surface and once on a foam surface (Figure 1). We counterbalanced the blocking of surface type (firm first and foam second vs. foam first and firm second), and we randomized all cognitive tasks within each block. At the end of the study, participants completed a survey before we debriefed and compensated them.

### 2.3. tDCS Procedure

**Figure 2:**
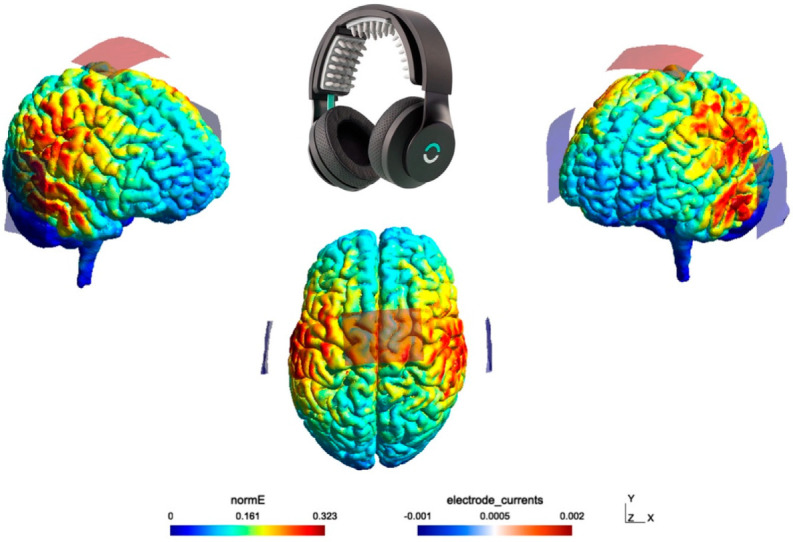
Modeled stimulation electric field along with Halo Sport headset using SimNIMBS v3.2.5.

We delivered non-invasive tDCS via a commercial off-the-shelf device called the Halo Sport [47]. The headset contained 4 cm × 6 cm electrodes that were soaked with saline before being placed on the scalp to help ensure an uninterrupted flow of current. The nominal contact regions were approximately 28 cm^2^, and the current density at the stimulation electrodes was 0.071 mA/cm^2^, which is well below that which has been shown to damage brain tissue [48] and is within the range of what has been used in prior research [49]. After an initial minute of stimulation, we had participants rate their level of sensation in terms of pain, itchiness, heat, and overall discomfort on an 11-point scale (with 0 indicating no feeling at all and 10 indicating extreme sensation [50,51]) and asked them if they were comfortable proceeding with the study. From that point, the active tDCS condition consisted of approximately 40 min of continuous stimulation at 2 mA over the primary motor cortex, corresponding to electrode Cz (based on a the 10–20 system) for anodal and C5/C6 for cathodal, which Halo classifies as “legs, core, arms” (Figure 2). These parameters are consistent with prior motor cortex stimulation research [52]. The sham tDCS condition used the same electrodes but only delivered current briefly at the start and end of the session similar to prior research [53]. Participants and experimenters were blinded to tDCS condition, which was achieved via the Halo Sport Researcher Portal on the computerized tablets. Specifically, a 3rd party not involved in data collection or analysis randomly assigned half of the participant IDs to receive active stimulation and half to receive sham stimulation, and this stimulation assignment remained blinded throughout the study duration. Furthermore, the software used to administer the current appeared identical across active and sham conditions to prevent those administering the current from knowing what kind of stimulation was being delivered.

### 2.4. Balance Procedure

We used six APDM Opal IMUs to measure postural sway (Opal v2, APDM Inc., Portland, OR, USA). We placed these sensors on the wrists, feet, sternum, and lumbar [54,55,56,57,58,59]. This allowed us to measure the center of pressure (COP) variability via the root mean square distance of sway acceleration (RMS Sway in m/s^2^), which quantifies the magnitude of center of pressure displacements [60,61,62,63]. We used a foot placement template to ensure consistent foot placement across all trials, such that participants had roughly 10 cm between the left and right heel with a 30-degree outward foot rotation (Chiari 2002; Morris 2019). For the stable conditions, participants stood on the firm tile floor of the lab. For the unstable conditions, participants stood on an Airex Elite foam balance pad approximately 6 cm in height and commonly used in postural control research [64,65]. In baseline trials, we instructed participants to simply stand while holding and looking at a tablet. In dual-task trials, we instructed participants to complete cognitive tasks via tablets while standing (Figure 1). We used APDM Mobility Lab v2.0 to process the postural sway data [66].

### 2.5. Computerized Cognitive Tasks

We administered tablet-based cognitive tasks via the mobile application BrainBaseline, which is a scientifically validated research tool used with both young and older adults [44,67,68].

For our non-EF measure, we used a simple processing speed task [69]. In this task, participants were instructed to respond as quickly as possible whenever a circle appeared in the middle of the screen, and we used response time as our primary measure. 

For our EF measures, we used a shifting task, an updating task, and an inhibition task to capture the three broad constructs reported in other work [70]. In the EF shifting task, participants classified a number as either odd vs. even or greater than vs. less than 5, depending on the background color on each trial, and we calculated switch costs (i.e., the difference between correct switch trial RTs and correct repeat trial RTs) as our primary measure [71]. 

In the EF updating task (N-Back), participants viewed a stream of numbers presented sequentially and had to determine whether the current number matched the number presented two trials previously, and we used 2-back accuracy as our primary measure [72]. 

In the EF inhibition task (Stroop), participants responded to the font color of centrally presented words while ignoring the lexical content of the word, and we calculated the Stroop effect (i.e., difference between correct incongruent RTs and correct congruent RTs) as our primary measure [73,74]. Full details on all four tasks can be found in the appendix of [67].

### 2.6. Data Analysis

The wearable sensors we used were incredibly sensitive to very small movements. Thus, we took several steps in preparing the data prior to analysis. First, we trimmed five seconds from the start and end of each recording because we found that participants had excessive movement as they started and ended each trial. We attributed this to the fact that they turned toward the experimenter at the beginning and end of a trial for instructions. Next, we flagged and removed outlier participants whose postural sway data were 1.5 times beyond the interquartile range [75], which resulted in a final sample size of 52 participants (27 active stimulation and 25 sham stimulation). We subsequently restricted our cognitive task analysis to these same 52 participants so that there was a direct correspondence between our balance performance analysis and cognitive performance analysis. According to a sensitivity analysis in G*Power 3.1, we should have been able to detect reliable between-subjects effects for F values at or above 4.03, as well as within-subjects effects for F values at or above 1.90 [76,77]. 

For the postural sway data, we conducted a 2 (stimulation: active vs. sham) × 5 (cognitive load: baseline vs. processing speed vs. EF shifting vs. EF updating vs. EF inhibition) × 2 (surface stability: firm vs. foam) mixed-model ANOVA. For the cognitive data, we conducted a series of mixed-model ANOVAs for the different dependent cognitive measures with surface stability as a 2-level within-subjects factor and stimulation as a 2-level between-subjects factor. We used jamovi (v1.6) for all analyses and applied Greenhouse–Geisser corrections when sphericity was violated. The significance level was set a priori at *p* < 0.05.

## 3. Results

### 3.1. Balance Performance

**Figure 3:**
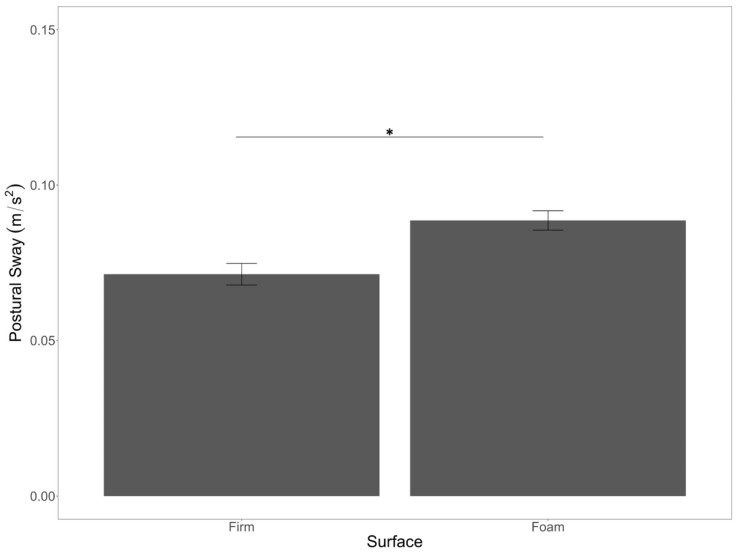
Main effect of surface stability on postural sway. Error bars are +/− SE. Significant differences are indicated by asterisks: * *p* < 0.05.

For the postural sway data, we conducted a 2 (stimulation: active vs. sham) × 5 (cognitive load: baseline vs. processing speed vs. EF shifting vs. EF updating vs. EF inhibition) × 2 (surface stability: firm vs. foam) mixed-model ANOVA (see Supplementary Materials for postural sway descriptive and inferential statistics). There was a main effect of surface stability (*F*(1,50) = 34.74, *p* < 0.001, *η*^2^*_p_* = 0.41) with significantly lower sway on the firm surface compared to the foam surface (Figure 3), which aligned with our predictions. 

**Figure 4:**
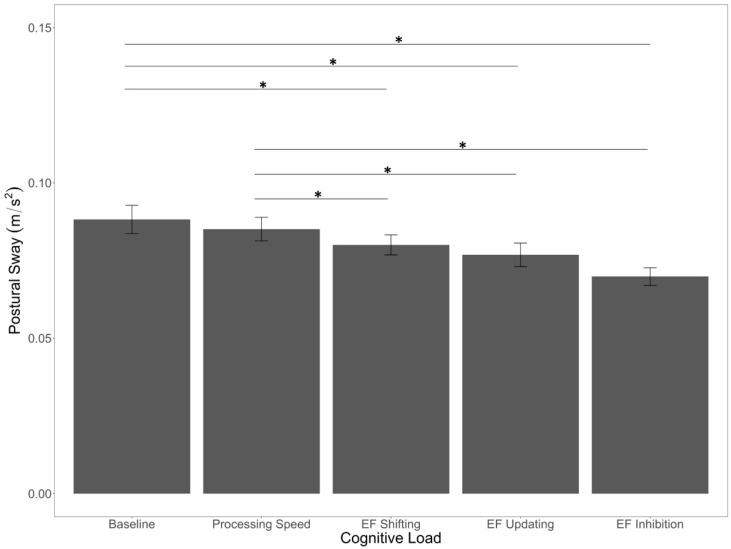
Main effect of cognitive load on postural sway. Error bars are +/− SE. Significant differences are indicated by asterisks: * *p* < 0.05.

Additionally, there was a main effect of cognitive load (*F*(4,200) = 8.49, *p* < 0.001, *η*^2^*_p_* = 0.15). Counter to our predictions, planned comparisons revealed that postural sway was significantly higher in the baseline condition compared to the EF shifting (*p* = 0.02), EF updating (*p* = 0.04), and EF inhibition (*p* < 0.001) conditions. Postural sway did not differ between the baseline and processing speed conditions (*p* = 0.41), although the EF conditions all had significantly lower postural sway compared to the processing speed condition (*p*’s < 0.05; Figure 4). 

**Figure 5:**
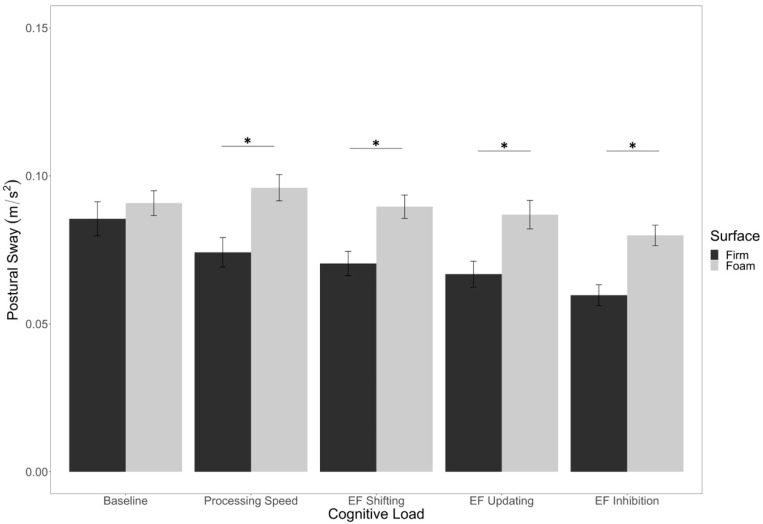
Interaction of surface stability and cognitive load on postural sway. Error bars are +/− SE. Significant differences are indicated by asterisks: * *p* < 0.05.

In addition, there was a significant interaction between surface stability and cognitive load (*F*(4,200) = 2.61, *p* = 0.04, *η*^2^*_p_* = 0.05), in which postural sway in the firm condition was significantly lower than the foam condition for all levels of load (*p*’s < 0.05) except the baseline (*p* = 0.22; Figure 5). 

**Figure 6:**
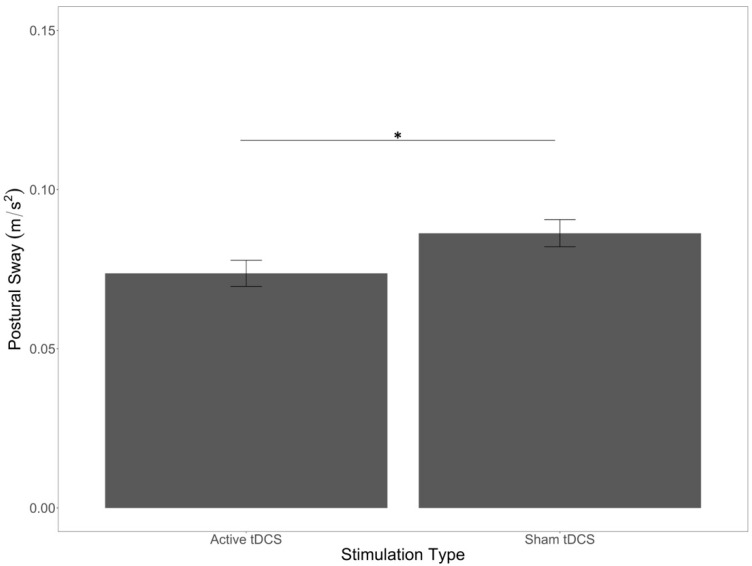
Main effect of stimulation type on postural sway. Error bars are +/− SE. Significant differences are indicated by asterisks: * *p* < 0.05.

Finally, we found a main effect of stimulation (*F*(1,50) = 4.53, *p* = 0.04, *η*^2^*_p_* = 0.08) in which postural sway was significantly lower in the active stimulation group compared to the sham stimulation group (Figure 6). Stimulation did not interact with any other factor, nor were there any other significant interactions among the factors.

### 3.2. Cognitive Performance

For the cognitive data, we conducted a series of mixed-model ANOVAs for the different dependent measures with surface stability as a 2-level within-subjects factor and stimulation as a 2-level between-subjects factor (see Supplementary Materials for cognitive task descriptive and inferential statistics). 

In terms of processing speed, we found no effect of surface stability (*F*(1,50) = 1.45, *p =* 0.23, *η*^2^*_p_* = 0.03), no effect of stimulation (*F*(1,50) = 0.05, *p* = 0.82, *η*^2^*_p_* = 0.001), and no interaction between these factors (*F*(1,50) = 2.07, *p* = 0.16, *η*^2^*_p_* = 0.04). 

In terms of EF shifting, we found no effect of surface stability (*F*(1,50) = 0.79, *p* = 0.38, *η*^2^*_p_* = 0.02), no effect of stimulation (*F*(1,50) = 2.24, *p* = 0.14, *η*^2^*_p_* = 0.04), and no interaction between these factors (*F*(1,50) = 0.99, *p* = 0.32, *η*^2^*_p_* = 0.02). 

In terms of EF updating, we found no effect of surface stability (*F*(1,50) = 0.32, *p* = 0.58, *η*^2^*_p_* = 0.01), no effect of stimulation (*F*(1,50) = 0.11, *p* = 0.75, *η*^2^*_p_* = 0.002), and no interaction between these factors [*F*(1,50) = 1.07, *p* = 0.31, *η*^2^*_p_* = 0.02]. 

In terms of EF inhibition, we found no effect of surface stability (*F*(1,50) = 1.42, *p* = 0.24, *η*^2^*_p_* = 0.03), no effect of stimulation (*F*(1,50) = 0.41, *p* = 0.53, *η*^2^*_p_* = 0.01), and no interaction between these factors (*F*(1,50) = 1.07, *p* = 0.31, *η*^2^*_p_* = 0.02). 

To summarize, there were no significant main effects of surface stability or stimulation or interaction between these factors in our cognitive data.

### 3.3. Post Experiment Survey

At the end of the study, we had the participants complete a survey that included several questions about their experience during the study. For example, we asked participants in both stimulation groups if they noticed the stimulation and if the stimulation distracted them. Groups did not differ in terms of noticing the stimulation (*χ*^2^(df = 1, N = 52) = 0.66, *p* = 0.42) nor in terms of being distracted by the stimulation (*χ*^2^(df = 1, N = 52) = 2.92, *p* = 0.09). 

We also asked participants at the end of the session to provide levels of different scalp sensations, such as pain, discomfort, itching, and burning [78]. Although the stimulation groups did not differ in terms of itching sensation (*t*(50) = 0.87, *p* = 0.39, *Cohen’s d* = 0.24), the active tDCS group reported higher sensations in terms of pain (*t*(50) = 2.54, *p* = 0.01, *Cohen’s d* = 0.70), discomfort (*t*(50) = 2.32, *p* = 0.03, *Cohen’s d* = 0.64), and burning (*t*(50) = 3.23, *p* = 0.002, *Cohen’s d* = 0.90). 

Finally, we asked the participants to guess which stimulation condition they were in using a sliding scale, with the lower anchor being “1: I definitely received 40 min of stimulation” and the upper anchor being “11: I definitely received 1 min of stimulation”. Here, too, the groups differed (*t*(50) = 6.03, *p* < 0.001, *Cohen’s d* = 1.67(, with the active tDCS group reporting an average of 2.59 (SD = 2.15) and the sham tDCS group reporting an average of 6.44 (SD = 2.45).

## 4. Discussion

In this study, we investigated the impact of tDCS on postural sway while participants stood on stable and unstable surfaces and completed single-task trials, as well as dual-task trials. The dual task conditions included a processing speed measure and three executive function measures (i.e., shifting, updating, and inhibiting). We predicted that postural sway would be greater on unstable surfaces, and that is exactly what we found with greater sway in the foam surface compared to the firm surface, which aligns with prior research [17,18].

We also predicted an impact of cognitive load. Specifically, we thought that dual-task situations (i.e., when performing cognitive tasks) would lead to greater sway compared to a baseline control condition. We did find an overall effect of cognitive load; however, the single-task baseline condition had significantly more sway than the EF conditions. In other words, the effect was in the opposite direction from what we predicted. We based our initial prediction on prior research that has found greater sway during dual-task balance scenarios [79,80], but it is important to note that others have found decreases in postural sway during cognitive–motor multitasking [81,82,83]. In fact, some have argued that there are at least nine different patterns of results one might expect when investigating cognitive–motor multitasking [84]. The present data suggest that the heterogeneity of sway-related outcomes is not reliably related to the nature of the EF task employed, though EF demands did generally reduce sway relative to a non-EF processing speed condition. It is unclear why EF demands reduced sway, though it could indicate an advantage of relatively automatic (rather than intentional) postural control processes that are active when attention is allocated to external tasks [85]. For example, when participants are asked to focus their attention on maintaining postural stability on an uneven surface, they show *more* postural sway than when they are not provided such instructions [86]. It could be the case that the extent of attention allocated to an external task—for example, EF versus processing speed demands—is associated with an increase in relatively natural postural control processes. These relatively natural control processes might be sufficient for maintaining posture while on a surface that is only minimally uneven and unchanging, like the one used at present. Of course, this does not explain why some studies find evidence for cognitive tasks reducing postural stability, particularly as these tasks become more demanding [79,87]. Further research is needed to better understand when secondary cognitive tasks lead to increased sway vs. decreased sway. 

Regarding the effect of tDCS over primary motor cortex, we predicted an impact of stimulation on sway. Specifically, we thought that active tDCS would reduce postural sway compared to sham tDCS, and this is what we observed. This is similar to prior work with healthy young [32] and older adults [88,89], except that our study is the first to show this with tDCS over motor cortex rather than dorsolateral prefrontal regions in the context of cognitive–motor dual-tasking. In addition, we used multiple cognitive tasks as opposed to a single serial subtraction task. That said, the impact of tDCS was limited to a main effect, and we did not find any interactions between tDCS and our other factors of cognitive load and surface stability. The anodal stimulation of the primary and supplemental motor areas may increase neural plasticity and facilitate motor learning [90,91], although more research is needed to test this in acute settings. In the present study, increased neuroplasticity in these regions may have accelerated the retention and implementation of simple postural adaptations learned by the cerebellum in response to the uneven surface [92]. Continuing research may benefit from understanding how prefrontal, motor cortical, and cerebellar stimulation may interact with the ability to sustain performance with concurrent cognitive and motor demands in cognitive–motor dual-task settings, as well as with additional clinical populations [68]. 

On a related note, we also had participants reflect on their experience at the end of the study. Our tDCS groups did not differ in terms of noticing the stimulation or being distracted by the stimulation. However, we also asked participants to what degree they experienced different scalp sensations (i.e., pain, discomfort, itching, and burning) on a scale from 1 (none) to 11 (unbearable). Our active tDCS group reported higher scalp sensations in terms of pain, discomfort, and burning (but not itching) relative to the sham group. It is possible that this impacted our subsequent question about stimulation group assignment. Specifically, we asked participants to guess which condition they were in using a scale from 1 (“I definitely received 40 min of stimulation”) to 11 (“I definitely received 1 min of stimulation”). Participants in the active tDCS group had significantly lower ratings than those in the sham tDCS group, which could have resulted from the greater scalp sensations in the active tDCS group. In general, this pattern of results could suggest that the active tDCS groups were not fully blinded to their tDCS condition assignment, and this could have impacted their cognitive–motor multitasking performance, akin to a placebo effect [93,94,95], although correctly guessing stimulation condition does not necessarily alter the effects of tDCS on performance [96]. 

### 4.1. Limitations and Future Directions

Our study has several additional limitations that can serve as opportunities for future research. For example, our tDCS blinding may have been ineffective, since participants in the active tDCS group seemed to correctly guess they had received active tDCS compared to the sham tDCS group. This is not without precedent in the literature, especially at higher levels of stimulation [97], and it indicates a clear need for better tDCS blinding procedures to avoid any confounding impact of expectations [98], although at least one study found no clear effects of expectations on performance [99]. 

In addition, we also had the baseline single-task condition first and then counterbalanced the subsequent firm and foam conditions across participants. It is possible that this could have led to order effects, which future research could avoid by counterbalancing or randomizing all experimental conditions. On a related note, we did not include seated control conditions for the cognitive tasks in part because we were concerned about a lengthy experimental session giving rise to participant fatigue. That said, future studies could include seated versions of the cognitive tasks and different types of cognitive tasks, which would allow for directly comparing cognitive costs in addition to motor costs.

As mentioned in our Methods section, we chose to measure postural sway with wearable sensors in part because of past research indicating their reliability and sensitivity [54,57,63]. Unfortunately, we ran into significant technical difficulties which resulted in either data loss or significantly outlying data for 28 of our 80 participants. Future studies with larger samples could consider novel data cleaning methods, as well as leverage converging evidence from alternative postural sway measurement approaches (i.e., motion capture or force plates) when feasible. That said, some of the advantages of using wearable sensors are that they are easy to set up, affordable, and portable, which could increase access to special populations that would not normally come to the lab for studies (i.e., community-based samples; [68]).

Finally, it is important to note that the present work constitutes the first instance where the Halo Sport, a commercially available neurostimulation device, was used to impact cognitive–motor multitasking. We used a standard montage offered by Halo to target the primary motor strip. The device’s form factor takes on a headphone model to maximize a comfortable design during physical activity. It is possible that the device may have been placed non-uniformly on participants’ heads, leading to the possibility that different brain regions were inadvertently targeted across our sample. In addition, it is unclear if this device could be used for anything other than motor cortex stimulation given the fixed form factor. This is unfortunate given that prior studies with research-grade tDCS devices have found promising results when stimulating more frontal areas in cognitive–motor multitasking paradigms [32,33]. Thus, future cognitive–motor multitasking work could benefit from a comparison of Halo Sport with research-grade tDCS devices to explore these possibilities.

### 4.2. Conclusions

In modern society, humans rarely stand upright on completely stable surfaces and without additional cognitive tasks. While there is mounting evidence that cognitive tasks impact balance, less is known about the specificity of secondary cognitive task impact on balance. Furthermore, there has been increased interest in leveraging non-invasive brain stimulation (e.g., tDCS) to mitigate the effects secondary cognitive tasks have on balance. In the current study, we used a commercially available tDCS device. We also used both EF tasks and a non-EF control task and found that EF tasks impacted balance more than the non-EF task, albeit in a pattern counter to what we had anticipated. Interestingly, tDCS did not interact with the cognitive task type; however, tDCS in general did lead to reductions in cognitive–motor multitasking costs (i.e., less sway). Due to the increasing role devices play in daily life, we used tablet-based cognitive tasks similar to other studies that found impacts of mobile devices on balance for both younger and older adults [18,19,100,101,102,103]. Future work is needed to investigate how our results may extend to special populations for whom balance might be impaired, as well as to interventional contexts that leverage other cognitive enhancement measures, such as cognitive training and physical activity, in addition to non-invasive brain stimulation [23]. 

## Data Availability

The data presented in this study are available upon reasonable request from the corresponding author.

## Supplementary Materials

The following are available online at https://www.mdpi.com/article/10.3390/brainsci12020180/s1, Table S1: Postural sway descriptive statistics, Table S2: Cognitive task descriptive statistics, Table S3: Inferential statistics for balance performance, Table S4: Inferential statistics for cognitive performance.
