# Supplementary material for: Modulating Cognitive–Motor Multitasking with Commercial-off-the-Shelf Non-Invasive Brain Stimulation
Source: Brain Sci. 2022 Jan 29;12(2):180. doi: 10.3390/brainsci12020180 (PMC8870640; doi:10.3390/brainsci12020180)
Supplement: Supplementary file 1 [file brainsci-12-00180-s001.zip › brainsci-1576145-supplementary.pdf]

Table S1. Postural sway descriptive statistics.

		Stimulation Type			
		**Active**		**Sham**	
**Surface**	**Cognitive Load**	Mean	SD	Mean	SD
Firm	Baseline	0.084	0.045	0.100	0.044
	Processing speed	0.075	0.035	0.084	0.039
	EF Shifting	0.077	0.036	0.079	0.039
	EF Updating	0.066	0.024	0.074	0.037
	EF Inhibition	0.058	0.021	0.063	0.028
Foam	Baseline	0.090	0.033	0.101	0.034
	Processing speed	0.103	0.045	0.118	0.052
	EF Shifting	0.091	0.030	0.098	0.034
	EF Updating	0.089	0.033	0.099	0.045
	EF Inhibition	0.079	0.028	0.086	0.026

Table S2. Cognitive task descriptive statistics.

		Stimulation Type			
		**Active**		**Sham**	
**Surface**	**Cognitive Load**	Mean	SD	Mean	SD
Firm	Processing speed	328	41	335	33
	EF Shifting	186	122	130	93
	EF Updating	0.90	0.09	0.92	0.09
	EF Inhibition	144	112	117	80
Foam	Processing speed	336	45	334	31
	EF Shifting	184	129	164	91
	EF Updating	0.92	0.08	0.91	0.08
	EF Inhibition	115	79	115	69

Table S3: Inferential statistics for balance performance

	F	df	p	η^2_p
Surface Stability	34.74	1,50	< .001	0.41
Cognitive Load	8.49	4,200	< .001	0.15
Surface Stability x Cognitive Load	2.61	4,200	0.04	0.05
Stim	4.53	1,50	0.04	0.08
Stim x Surface Stability	0.00	1,50	0.99	0.00
Stim x Cognitive Load	1.51	4,200	0.20	0.03
Stim x Surface Stability x Cognitive Load	0.67	4,200	0.62	0.01

Table S4: Inferential statistics for cognitive performance

		F	df	p	η^2_p
Non-EF Processing Speed	Surface Stability	1.45	1,50	0.23	0.03
	Stim	0.05	1,50	0.82	< .001
	Stim x Surface Stability	2.07	1,50	0.16	0.04
EF Shifting	Surface Stability	0.79	1,50	0.38	0.02
	Stim	2.24	1,50	0.14	0.04
	Stim x Surface Stability	0.99	1,50	0.32	0.02
EF Updating	Surface Stability	0.32	1,50	0.58	0.01
	Stim	0.11	1,50	0.75	< .001
	Stim x Surface Stability	1.07	1,50	0.31	0.02
EF Inhibition	Surface Stability	1.42	1,50	0.24	0.03
	Stim	0.41	1,50	0.53	0.01
	Stim x Surface Stability	1.07	1,50	0.31	0.02
